# Flexible Surface Acoustic Wave (SAW) Magnetic Sensor Based on Terfenol-D Grating-Arrayed Thin Polymer Film

**DOI:** 10.3390/mi17050537

**Published:** 2026-04-28

**Authors:** Akeel Qadir, Fayyaz Muhammad, Shahid Karim, Jinkai Chen, Hongsheng Xu, Umar Farooq

**Affiliations:** 1 School of Information Engineering, Xi’an Eurasia University, Xi’an 710065, China; 2 Electronics Division, Pakistan Institute of Nuclear Science and Technology (PINSTECH), Islamabad 45650, Pakistan; 3 Ministry of Education Key Laboratory of RF Circuits and Systems, Hangzhou Dianzi University, Hangzhou 310018, China; chenjk09@hdu.edu.cn; 4 Industry-Education-Research Institute of Advanced Materials and Technology for Integrated Circuits, Anhui University, Hefei 230601, China; 5 Department of Mechanical Engineering, University of Colorado Boulder, Boulder, CO 80309, USA

**Keywords:** Surface Acoustic Wave sensors, magnetic field sensors, thin film, microelectromechanical (MEMS) sensors

## Abstract

Surface Acoustic Wave (SAW) magnetic sensors are traditionally fabricated on rigid substrates, which severely limits their application on curved or irregular surfaces. To address this critical limitation, this paper presents a novel flexible SAW magnetic sensor based on a grating-arrayed Terfenol-D thin film deposited on a 50 µm thick flexible lithium niobate (LiNbO_3_) substrate. Unlike conventional designs using a continuous magnetostrictive layer, the proposed grating-arrayed structure is designed to aid in hysteresis compensation and minimize measurement errors associated with residual magnetization. As demonstrated experimentally, the sensors achieve a high sensitivity of 85.8 kHz/mT for devices with λ-wide gratings and a maximum frequency shift of 377 kHz at 5 mT. A systematic investigation reveals that sensitivity is critically dependent on the grating width and film thickness, with 500 nm thick gratings yielding optimal performance. Crucially, the sensor’s functionality under mechanical deformation is validated, and a differential measurement method is introduced to effectively compensate for stress-induced frequency shifts, ensuring reliable operation in practical, non-ideal conditions. The results confirm the sensor’s robust performance under the tested stress conditions, positioning this flexible SAW magnetic sensor as a promising solution for advanced, conformable sensing applications.

## 1. Introduction

In recent years, the burgeoning interest in flexible devices is attributable to their exceptional ability to maintain high performance levels while enduring mechanical deformations such as bending and stretching. This advancement marks a pivotal shift in device technology, heralding a new era where flexibility and efficiency coexist seamlessly [1,2]. These characteristics enable these devices to become a part of many applications that require the device to fit on irregular and soft surfaces or objects where traditional devices fabricated on rigid wafers do not work [3]. Real-time monitoring plays a crucial role across diverse sectors, including manufacturing industries, civil infrastructure, aerospace engineering, and the automotive industry, serving as an indispensable tool for health monitoring and failure prevention [4]. The global potential of flexible sensors has ignited a fervent pursuit in both scientific and industrial realms. This collective effort is driven by an unwavering demand for robust, exquisitely sensitive, and flexible sensor technologies capable of seamlessly integrating with diverse and ever-evolving environments. Evolutionary, the progression in lithography technologies has made incredible improvement in the development of flexible and stretchable electronics, providing a new exciting functionality over conventional stiff substrates [5,6].

Surface Acoustic Wave (SAW) devices are popular for their compelling qualities such as wireless passive operations, a flexible substrate, compact size, etc., and they have been widely investigated for applications such as frequency filters, microelectron systems, and microfluidics. The inspiring benefits of flexible technology and advancement in manufacturing processes and innovative methods have significantly stimulated the research of flexible SAW devices. SAW devices are widely used for biomedical applications and sensor systems [7,8]. Several flexible SAW sensors have been developed and reported in the literature [9,10,11]. However, SAW magnetic sensors are normally fabricated on rigid substrates. Despite the progress in flexible magnetic sensors, there is an obvious lack in the development of flexible Surface Acoustic Wave (SAW) magnetic sensors. Addressing this gap by integrating flexibility into SAW magnetic sensor design is crucial, ensuring compatibility with modern flexible systems and broadening their application scope. Therefore, flexible SAW magnetic sensor design became the main focus of research and experimental work, in order to present a new concept in the designing of the SAW magnetic sensor and demonstrate its practicality [12,13,14,15,16].

SAW magnetic sensors vary in design and methods of measurements [17,18]. Generally, they are classified into three kinds; magnetostrictive thin-film-based sensors, GMI-based sensors and one-port resonant sensors. In this paper, a novel idea of flexible SAW magnetic sensor design is discussed in detail. The proposed new sensor design is based on a thin film of Terfenol-D (Tb0.3 Dy0.7 Fe1.92) magnetostrictive material deposited in the delay line of a two-port SAW device fabricated on a flexible Lithium Niobate LiNbO_3_ substrate. The design follows the new and growing trend of developing flexible SAW devices and is inspired by earlier work related to magnetostrictive TbDyFe alloy deposition on flexible substrates for micro devices [19]. The flexible SAW magnetic sensors that are presented in this paper are based on Terfenol-D (Tb0.3 Dy0.7 Fe1.92), which is a well-known magnetostrictive material and has been used in many industrial applications because of its large saturation magnetostriction and notable magnetic properties [20,21]. Due to its exclusive magnetoelastic properties, this material has been selected to be used in a flexible SAW magnetic sensor as a sensing element. The rest of the paper is organized as follows: Section 2 describes the design layout and working of the proposed sensor, Section 3 briefly covers the details regarding the fabrication and prototyping of the sensors. Experiment details, results obtained and analyses are presented in Section 4.

## 2. Fabrication and Device Mechanism

### 2.1. Mechanism

**Figure 1:**
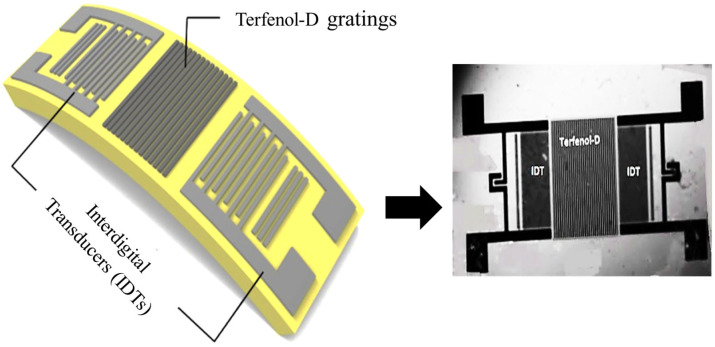
Device-2 schematic and microfabrication. The schematic of Device-2 visualized by the Terfenol-D gratings deposited between the delay line.

Unlike conventional designs, the proposed design uses a thin film of Terfenol-D in the form of gratings instead of a continuous patch of thin film of magnetostrictive material as shown in Figure 1. This design method aids in reducing the hysteresis effect which is induced in magnetostrictive materials and causes measurement errors. It also contributes well to generating magnetostrictive strain [22]. When the electrical signal is applied at one of the Interdigital Transducers (IDT) serving as the input port of the SAW magnetic sensor, the stresses are generated because of the piezoelectric effect resulting in the generation of Surface Acoustic Waves (SAW) which propagate along the surface of the substrate. The traveling SAWs interact with the deposited Terfenol-D film in the delay line and reach another IDT serving as an output port. The mathematical equation of motion of these waves in piezoelectric LiNbO_3_ and non-piezoelectric media is given as [23]:

(1) $$\left\{\begin{array}{c} \rho_{s}\frac{\partial^{2}u_{i}^{I}}{\partial t^{2}}-\;c_{ijkl}^{I}\frac{\partial^{2}u_{k}^{I}}{\partial x_{l}\partial x_{j}}-e_{kij}\frac{\partial^{2}\Phi }{\partial x_{k}\partial x_{j}}=0\\ e_{jkl}\frac{\partial^{2}u_{k}^{I}}{\partial x_{l}\partial x_{j}}-\;\varepsilon_{jk}\frac{\partial^{2}\Phi }{\partial x_{k}\partial x_{j}}=0 \end{array}\right.$$

where Einstein’s summation rule is used, and the indices change from 1 to 3. In (1), Φ denotes the electric potential and $u_{i}^{I}$ represents the mechanical displacements. $e_{jkl}$,$\;c_{ijkl}^{I}$, and $\varepsilon_{jk}\;$ stand for the piezoelectric, elastic and dielectric constants respectively, and *ρ_s_* for the mass density of the piezoelectric substrate. Meanwhile, the wave equation in the isotropic and non-piezoelectric film (Terfenol-D) is given by:

(2) $$c_{ijkl}^{II}\frac{\partial^{2}u^{II}k}{\partial x_{l}\partial x_{j}}=\;\rho_{F}\frac{\partial^{2}u^{II}i}{\partial t^{2}},i,j,k,l=1,2,3,$$

Here, $c_{ijkl}^{II}$ and $\rho_{F}$ are the stiffness constants and density of the film.

When an external magnetic field H is applied, the Terfenol-D film exhibits magnetostrictive strain λ(H), which results not only in geometrical deformation but also in a modification of its effective elastic properties (ΔE-effect). Consequently, the film parameters become field-dependent, leading to perturbations in thickness h, density ρ, and elastic constants $c_{ijkl}^{II}$, which directly influence acoustic wave propagation.

(3) h=ho(1−β2)ρL=ρo(1+β2)×(1−β2)×(1−β2)

where $\rho_{o}$ and $h_{o}$ are the density and the thickness of unperturbed Terfenol-D film gratings and *β* is the magnetostrictive coefficient of the film related to the external magnetic field.

In addition to geometrical variations, the applied magnetic field modifies the Young’s modulus $E^{\prime }$ of the Terfenol-D film (ΔE-effect), which leads to corresponding changes in the elastic stiffness coefficients $c_{ijkl}^{II}$ as expressed in (4). These field-dependent elastic perturbations play a dominant role in altering the SAW propagation characteristics.

(4) $$\left\{\begin{array}{c} c_{11}=\frac{E^{\prime }\times (1-\mu )}{\left(1+\mu )(1-2\mu \right)}\\ c_{12}=\frac{E^{\prime }\times \mu }{\left(1+\mu )(1-2\mu \right)}\\ c_{44}=\frac{E^{\prime }\times (1-2\mu )}{2(1+\mu )(1-2\mu )} \end{array}\right.$$

The above field-induced variations in density, thickness, and elastic constants directly perturb the propagation of the Surface Acoustic Wave. Substituting these field-dependent parameters into the governing wave Equations in (1) and (2), the SAW phase velocity V becomes a function of the applied magnetic field through the modified material constants. As a result, the relative change in velocity ΔV/V arises primarily from the variation in elastic coefficients $c_{ijkl}^{II}(H)$, with secondary contributions from density and geometrical changes. Since the operating frequency of the device is related to the acoustic velocity by f=V/λ, and the wavelength is fixed by the IDT geometry, the measured frequency shift directly follows as Δf/f=ΔV/V. Therefore, the applied magnetic field modulates the SAW velocity through magnetoelastic coupling, which is observed experimentally as a shift in the operating frequency of the sensor.

The detailed fabrication process of the Surface Acoustic Wave (SAW) magnetic sensors fabricated on flexible LiNbO_3_ using the grating-arrayed thin film of Terfenol-D is presented in the next section.

### 2.2. Fabrication and Prototype Development

**Figure 2:**
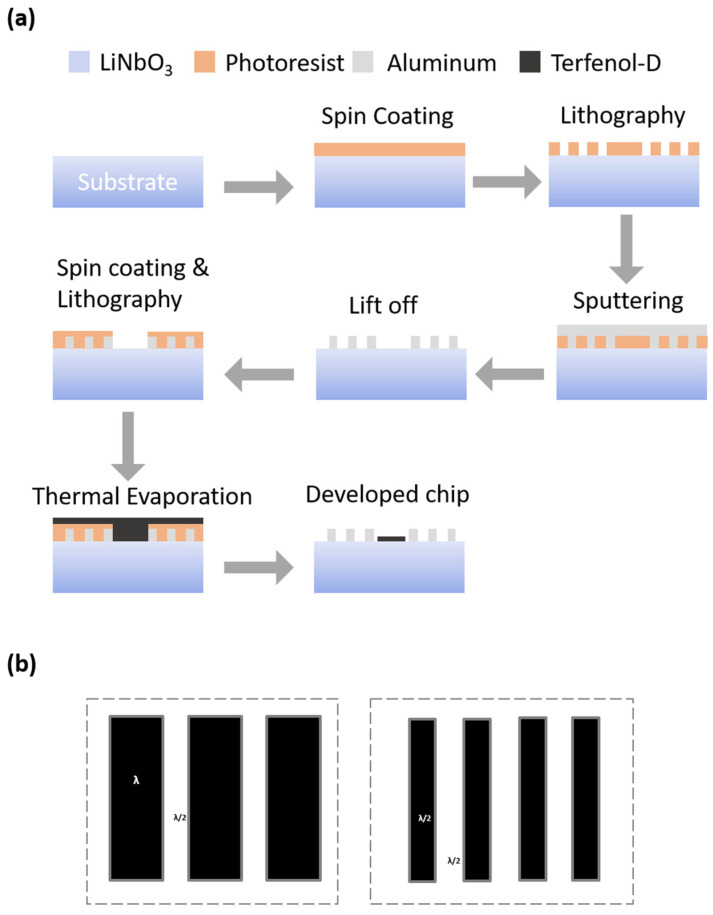
The microfabrication process and thin film grating. (**a**) Step-by-step microfabrication of SAW Device-2 and Terfenol-D thin film deposition process are shown. (**b**) The grating length spacing for Device-1 and Device-2 is illustrated. These varied designs were intended to be used to study the effects of film widths and thicknesses on the sensitivity of the magnetic sensors.

The proposed SAW magnetic sensor was fabricated on a 128o YX-cut flexible 50 µm thick Lithium Niobate LiNbO_3_ single-crystalline substrate similar to the one developed in [24]. The step-by-step microfabrication process is shown in Figure 2a. In the first stage, two-port SAW Device-2 was developed. First, the wafer surface was cleaned with a cleansing agent, Acetone Isopropyl deionized water, to remove impurities. This is to ensure that the metal deposits and the photolithography procedures do not encounter any manufacturing errors caused by inconsistencies from such impurities. Then a set of two Interdigital Transducers (IDTs) was developed on a LiNbO_3_ substrate using Aluminum (Al). This was achieved by depositing a uniform layer of Aluminum of ~200 nm thickness on the substrate using direct current (DC) magnetron sputtering (Atop Industry Co., Ltd, Jiangsu, China). The sputtering process was precisely optimized under carefully controlled conditions: a pressure of 0.5 Pa, a power setting of 200 W, and an Argon flow rate maintained at 100 sccm, ensuring precise and consistent material deposition. Subsequently, a layer of photoresist material was uniformly applied over the Aluminum metal layer, utilizing the spin coating technique. The desired Interdigital Transducer (IDT) pattern was accurately transferred onto the metal layer by utilizing a chrome photomask. Consequently, the photoresist layer underwent exposure to ultraviolet (UV) light using the MA6-BCA mask aligner (SUSS MicroTec, Garching, Germany). The Dry/Wet Etching process selectively removed the unprotected photoresist, exposing the underlying metal. This metal was then etched away while leaving behind the desired pattern protected by the photoresist. Subsequently, acetone was used to remove the protective photoresist layer. The wafer underwent an additional cleaning process to eliminate impurities. This step is essential before depositing the Terfenol-D thin film, an essential stage in converting the developed Surface Acoustic Wave (SAW) Device-2 into an effective magnetic sensor. A shadow mask was employed to cover the entire wafer, leaving exposed only the specific region nominated for the deposition of the thin film. The thin film gratings of Terfenol-D were then deposited in the desired portion using the DC magnetron sputtering process. The deposition parameters were 250 Watts of sputtering power, 3.0 mTorr of Ar pressure and wafer-to-target distance of 6.5 cm.

Two versions of these SAW magnetic sensors were produced with different widths of the thin film gratings as shown in Figure 2b. One of these sensor versions, Device-1, was designed with a grating width of λ and another sensor, Device-2, has a grating width of λ/2. The spacing between gratings was set to λ/2, where λ is the SAW wavelength of these devices.

**Figure 3:**
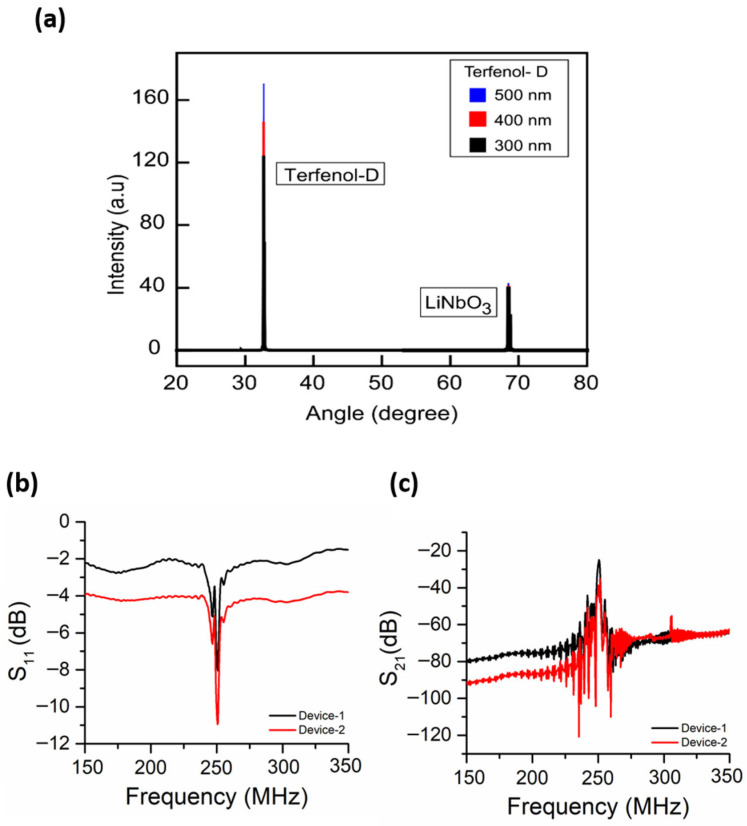
S-parameters characterizations; (**a**) X-ray Diffraction (XRD) results of devices with different thicknesses of Terfenol-D depositions. (**b**) The measured S-parameters of flexible SAW magnetic sensors; (**c**) S_11_ of Device-1 and 2 and (**c**) S_21_ of Device-1 and Device-2. To expose the SAW magnetic sensors to the magnetic fields for measurement purposes, the Helmholtz coil was used to generate the magnetic fields of magnitude ranging between ±5 mT.

Moreover, Terfenol-D film gratings with variable thicknesses of 300 nm, 400 nm and 500 nm were deposited in multiple models of these sensors, i.e., Device-1 and Device-2. These varied designs were intended to be used to study the effects of film widths and thicknesses on the sensitivity of the magnetic sensors. All these SAW magnetic sensors were designed to operate at a 250 MHz frequency with a corresponding wavelength of 15.968 μm. Each sensor comprised of 100 pairs of IDT electrodes and 100 grating strips of Terfenol-D in-between the IDTs. The aperture size and IDT spacing were set to 2 mm. The number of reflectors at each end was 10. After the fabrication, the developed sensors were examined to evaluate their quality of production. X-ray Diffraction (XRD) was performed to study the film structures of Terfenol-D. As seen in Figure 3a, XRD results show the Terfenol-D thin film deposition thickness. The developed sensors were then bonded using gold wires for electrical connectivity and integrated on flexible PCB boards for testing.

### 2.3. Experimental Setup

Before magnetic field measurements, the fabricated SAW sensors were characterized using a vector network analyzer (VNA) to obtain their S-parameters. The reflection and transmission responses were recorded in terms of S_11_ and S_21_ respectively. Figure 3b shows the measured $S_{21}$ and $S_{11}$ responses of Device-2. Devices exhibiting stable and well-defined transmission peaks were selected for subsequent magnetic field measurements. A Helmholtz coil was used to generate a uniform magnetic field in the range of ±5 mT for sensor characterization. All measurements were performed within this field range. The sensors were positioned at the center of the Helmholtz coils to ensure field uniformity, with the magnetic field aligned parallel to the sensitive axis of Terfenol-D (along the x-axis), as illustrated in Supplementary Materials Figure S2. The magnetic field at the sensor location was calibrated using a Hall-effect gaussmeter, and a current–field relationship was established prior to measurements.

The sensors under test were connected to the VNA to provide the input excitation signal and to record the transmission response. The VNA was interfaced with a computer via a GPIB connection, and data were acquired in real time using a LabVIEW program [25]. The LabVIEW (version 22.3) program was used to extract the operating frequency, defined as the frequency corresponding to the maximum magnitude of the $S_{21}$ response. The frequency shift was calculated as $\Delta f(H)=f(H)-f(H_{0})$, where $H_{0}$ is the reference field. To ensure repeatability, five devices of each type (Device-1 and Device-2) with different Terfenol-D thicknesses were measured individually.

The magnetic field was applied in both positive and negative directions, with its magnitude increased incrementally in steps of 0.5 mT. At each field step, the sensor was held for at least 1 min prior to measurement to ensure stabilization. During this period, 20 measurements were recorded and averaged to reduce noise and improve accuracy. This procedure minimizes measurement fluctuations and allows sufficient time for the magnetostrictive film to reach equilibrium after field-induced changes. To minimize hysteresis effects, each measurement cycle was preceded by a magnetization initialization step in which the magnetic field was first increased to near saturation and then returned to the starting value. All measurements were performed under a unidirectional field sweep unless otherwise specified.

## 3. Results and Discussions

**Figure 4:**
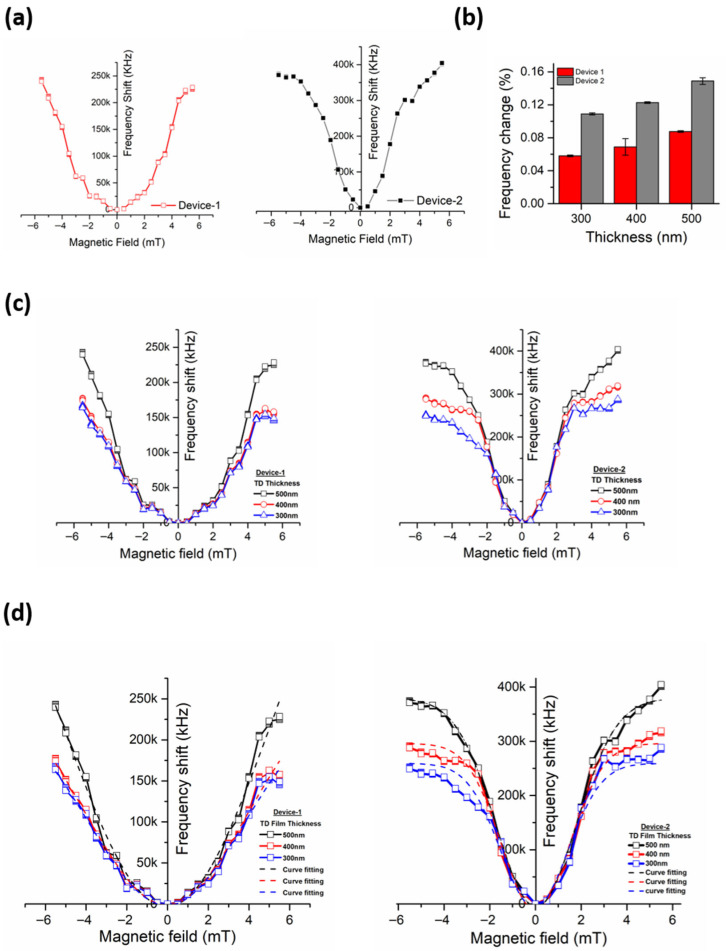
Terfenol-D Device-2’s characterization. (**a**) Frequency shift versus magnetic field in Device-1 and Device-2. (**b**) Percentage shift in frequencies versus film thickness. (**c**) Effect of film thicknesses on the measured frequency shifts of Device-1 and Device-2. (**d**) Curve fitting for Device-1 and Device-2 measured response curves.

The results reveal that the frequency shift in the sensors was proportional to the magnitude of the applied magnetic field. The measured responses were not ideally linear though, but they tended to attain linearity at lower field strengths because of the grating-arrayed thin film structure which helped in minimizing the effect of residual magnetization which is the major cause of non-linearity in measurements. The frequency shift observed was found to be different in both Device-1 and Device-2 due to the variable width of Terfenol-D gratings. The changes measured were larger in Device-1 sensors as compared to Device-2 sensors. The plot for Device-1 shown in Figure 4a is much steeper and linear than Device-2 due to an increase in frequency shift at a larger rate.

It is also noticed that besides grating widths, the grating thickness also contributed to the frequency shifts. The frequency shift between Device-1 and Device-2 is shown in Figure 4b. For the same magnetic field strength, the shift was higher for sensors with larger thicknesses of the gratings than that with lower thicknesses. The maximum change observed in Device-1 was in the sensor with the grating thickness of 500 nm and the corresponding shift in frequency was 377 kHz at 5 mT which was 0.15% of the actual frequency. Similarly, the maximum change in Device-2 was 223 kHz for the sensor with the grating thickness of 500 nm contributing to 0.089% of the change to the actual frequency. The frequency shift of Device-1 and Device-2 with different thickness is illustrated in Figure 4c.

The best curve fitting in Figure 4d was performed on each of the obtained curves for Device-1 and Device-2. The purpose was to determine the most suitable design of the sensor by comparing the measured response with the ideal estimated response plots. In both Device-1 and Device-2, Adjusted R Squared values were found higher for sensors with Terfenol-D film grating thickness of 500 nm than the sensors with film grating thicknesses of 300 nm and 400 nm. A complete tabulation of the adjusted $R^{2}$ and slope values for all device configurations are provided in the Supplementary Materials (Table S1). This implies that the response of the sensors with Terfenol-D film grating thicknesses of 500 nm was closer to the actual response with minimum errors. In addition, the steepness of the measured curves was calculated with the help of slopes. The slope of a curve defines the sensitivity of the sensors. The calculated slope values in the linear region of the curves were higher for sensors with a Terfenol-D film grating thickness of 500 nm than that of those with the thicknesses of 300 nm and 400 nm. Therefore, in the light of the obtained results, the rest of the experiments and analyses were carried out on Device-1 and Device-2 sensors with 500 nm thick Terfenol-D film gratings. Device-1 and Device-2 with 500 nm thick gratings of Terfenol-D have the highest sensitivity, which is 85.788 kHz/mT and 46.488 kHz/mT respectively in the magnetic field range of 0–5 mT. The same device showed a sensitivity of 84.124 kHz/mT and 43.261 kHz/mT for the reverse range of −5 mT and 0. The error in the sensitivity is due to the residual magnetization in Terfenol-D film gratings. Detailed statistical metrics, including standard deviation, coefficient of variation, and R^2^ analysis across all tested devices, are reported in the Supplementary Materials (Tables S3–S7).

Flexible sensors have the ability to measure despite their shape deformation. These sensors are primarily designed to be fixed on the rough and uneven surfaces where rigid sensors are not suitable. For a good flexible sensor, it is essential that its efficiency is not degraded even when it is bent during the measurement process. In order to qualify as a successful flexible sensor, the proposed flexible SAW magnetic sensor is required to prove its stability under different stress conditions. These stresses deform its structure which drastically changes the results obtained under normal conditions. Therefore, detailed analysis was needed to study the response of the flexible SAW magnetic sensor under different stress conditions and its impact on measurement results. Stress affects the magnetization of Terfenol-D as well as magnetostriction. Therefore, in many applications, Terfenol-D is pre-stressed before measurement in order to get the desired results. A quantitative comparison of key performance metrics with recent State-of-the-Art flexible SAW magnetic sensors is provided in the Supplementary Materials (Table S2).

The change in magnetostriction of Terfenol-D film gratings leads to the change in frequency shifts. Therefore, measurements by flexible SAW magnetic sensors are likely to be affected when stress is applied to them. The flexible SAW magnetic sensors (Device-1 and Device-2) were tested under variable stress conditions, designated as P1, P2, and P3, with P0 representing the unstrained reference state. The applied bending forces generated surface strains of 280 με, 550 με, and 730 με, respectively. Based on classical beam theory and the 50 μm substrate thickness, these strain levels correspond to bending radii of approximately 89.3 mm, 45.5 mm, and 34.2 mm. A detailed derivation of the strain-to-radius conversion is provided in the Supplementary Materials (Table S8). In order to measure these forces, the SAW magnetic sensor mounted on the flexible PCB board was bonded on a thin flexible metal plate equipped with a strain sensor as shown in Supplementary Materials Figure S2. The output of the strain was fed to the computer to measure the strain values. The bending stresses were applied to the metal plate with the help of a clamp.

**Figure 5:**
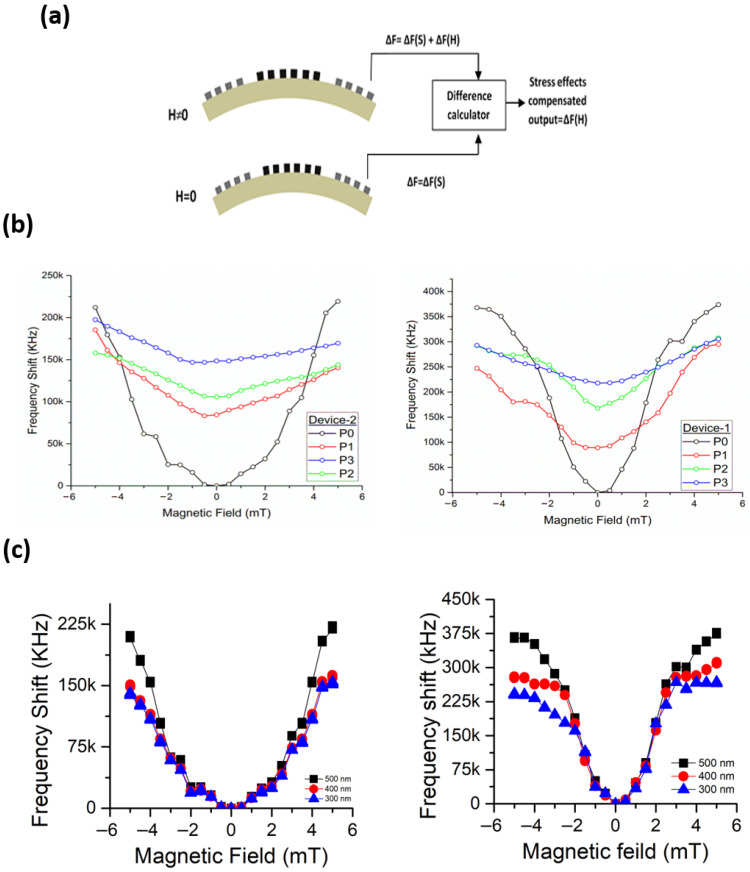
(**a**) Frequency offset compensation method. (**b**) Frequency shift vs magnetic field under different stress conditions. (**c**) Frequency offset compensated results of Device-1 and Device-2.

On the application of stress, the sensor mounted PCB board bent with the flexible metallic plate and the strain sensor recorded the amount of induced strain. The results obtained for Device-1 and Device-2 under these stress conditions are shown in Figure 5b.

In Figure 5b, it is notable that curves for different stress conditions have shifted upwards. When the stress was applied on the sensor it was bent to a certain degree. This bending introduced frequency shifts due to the strain produced as a result of substrate deformation. In addition, the magnetic film also experienced strain as a result of the applied stress. Both factors contribute to larger frequency shifts even at zero magnetic field. When the magnetic field was applied, the frequency was further shifted with the increment of the magnetic field at a slower rate. By applying the best curve fitting and slope calculations, it was found that for each value of the stress, the slope value is different. This implies that the sensitivity of the sensors Device-1 and Device-2 change under different stress conditions.

In order to accurately measure the frequency shifts with respect to the applied magnetic field, the offset in frequency shift due to the stresses must be compensated. One possible way to minimize this error is to take the differential output of the measurements taken at zero and non-zero magnetic field conditions. According to this approach, firstly the frequency shifts at zero magnetic field under different values of applied stresses are required to be measured. This will give the stress-dependent frequency shift values ΔF = ΔF(S) associated with the sensor being tested. Then the frequency shifts need to be measured at non-zero magnetic field conditions and applied stresses. This step will give the frequency shift values ΔF = ΔF(S) + ΔF(H) which will be the function of both the magnetic field and applied stresses. Hence, by taking the difference in measurements in step 1 and step 2, the effects of the applied stresses can be eliminated. The same procedure was adopted in the experiments and the differential output yielded the results which represent the frequency shifts due to the effects of the magnetic field only, i.e., ΔF = ΔF(H).

From the above figures, it is obvious that the results obtained by following the procedure described above have been notably compensated. The measured output in terms of observed frequency shifts now represents the actual results demonstrating the sensitivity of SAW magnetic sensors to the variable strength of the magnetic fields. Hence, the results acquired through experimental work have validated that the idea of developing a SAW magnetic sensor on a flexible substrate is a practical and reliable sensing solution. The flexible SAW magnetic sensors developed and used in the experiments have proved by their performance that these devices have the potential to sense the magnetic fields while undergoing mechanical deformation.

## 4. Conclusions

This research successfully introduces and validates a novel flexible SAW magnetic sensor fabricated on a 50 µm thick 128° YX-cut lithium niobate (LiNbO_3_) substrate. The sensor was implemented using a two-port delay line structure with an operating frequency of 250 MHz, where grating-arrayed thin films of Terfenol-D were deposited in the delay line between Interdigital Transducers (IDTs). Experimental characterization demonstrates that the sensor’s sensitivity is significantly influenced by the width and thickness of the Terfenol-D gratings. The optimal performance was achieved with 500 nm thick, λ-wide gratings (Device-1), which yielded a maximum sensitivity of 85.788 kHz/mT. Consistent with the design objective to improve upon rigid sensors, the grating-arrayed structure was designed to help mitigate hysteresis effects and minimize measurement errors associated with residual magnetization. While the measured response was not ideally linear due to the material’s inherent properties, the grating design facilitated a more predictable response and reduced errors. The developed sensors proved structurally robust, maintaining functionality under mechanical strains of up to 730 με. To ensure accuracy in conformable applications, a differential measurement method was introduced to effectively isolate magnetic frequency shifts from those induced by substrate deformation. Furthermore, the sensors demonstrated excellent thermal stability between 10 °C and 40 °C, with a negligible measurement error of only 1%. This work provides an encouraging foundation for future research aimed at enhancing sensitivity, improving accuracy, and validating long-term durability under cyclic mechanical loading.

## Data Availability

The original contributions presented in this study are included in the article/Supplementary Material. Further inquiries can be directed to the corresponding authors.

## Supplementary Materials

The following supporting information can be downloaded at: https://www.mdpi.com/article/10.3390/mi17050537/s1. Figure S1: Temperature-dependent characteristics. (a) Magnetization curve for Terfenol-D. (b,c) Frequency shift versus magnetic field under different temperature conditions. Figure S2: Experimental setup for testing SAW magnetic sensors. Figure S3: Illustration of the flexible SAW magnetic sensor under mechanical stress. Table S1: Summary of fitting statistics and sensitivity for Device-1 and Device-2. Table S2: Summary Table: Performance Metrics of Flexible SAW Magnetic Sensors. Table S3: Experimental statistics summary. Table S4: Sensitivity and statistical variation across devices. Table S5: Curve fitting quality (R^2^ Analysis). Table S6: Frequency stability and error analysis. Table S7: Repeatability across devices. Table S8: Applied strain and corresponding bending radius. References [26,27,28,29,30,31] are cited in supplementary file.
