# Supplementary material for: Flexible Surface Acoustic Wave (SAW) Magnetic Sensor Based on Terfenol-D Grating-Arrayed Thin Polymer Film
Source: Micromachines (Basel). 2026 Apr 28;17(5):537. doi: 10.3390/mi17050537 (PMC13208903; doi:10.3390/mi17050537)
Supplement: Supplementary file 1 [file micromachines-17-00537-s001.zip › micromachines-4262304-supplementary.pdf]

Flexible Surface Acoustic Wave (SAW) Magnetic Sensor Based on Terfenol-D Grating-Arrayed Thin Polymer Film

Akeel Qadir [1,†], Fayyaz Muhammad [2,†], Shahid Karim [1], Jinkai Chen [3], Hongsheng Xu [4,*] and Umar Farooq [5,*]

[1] School of Information Engineering, Xi'an Eurasia University, Xi'an 710065, China
[2] Electronics Division, Pakistan Institute of Nuclear Science and Technology (PINSTECH), Islamabad 45650, Pakistan
[3] Ministry of Education Key Laboratory of RF Circuits and Systems, Hangzhou Dianzi University, Hangzhou 310018, China; chenjk09@hdu.edu.cn
[4] Industry-Education-Research Institute of Advanced Materials and Technology for Integrated Circuits, Anhui University, Hefei 230601, China
[5] Department of Mechanical Engineering, University of Colorado Boulder, Boulder, CO 80309, USA
* Correspondence: xhs@ahu.edu.cn (H.X.); umar.farooq@colorado.edu (U.F.)
† These authors contributed equally to this work.

Supplementary material

Temperature dependent Characteristics:

The temperatures were varied between 10ºC and 40ºC. The measurement results of Device-1 and Device-2 under varying temperature conditions are shown in Figure S1. By applying the best curve fitting techniques and slope measurements the sensitivity of these devices corresponding to the particular temperature was obtained. For the temperature range from 10ºC to 40ºC, the sensitivity of Device-1 was deviated by up to 2.3 kHz and 3.54 kHz from the mean value within the magnetic field ranges 0 to +5mT and -5mT to 0 respectively. Moreover, the sensitivity of Device-2 was shifted up to 0.2 kHz and 0.050 kHz from the mean value within the magnetic field ranges 0 to +5mT and -5mT to 0 respectively. For both devices i.e. Device-1 and Device-2 these variations in the values of the sensitivity resulted in a measurement error of only 1%. This shows that temperature has minimal effects on the performance of the proposed sensor designs.

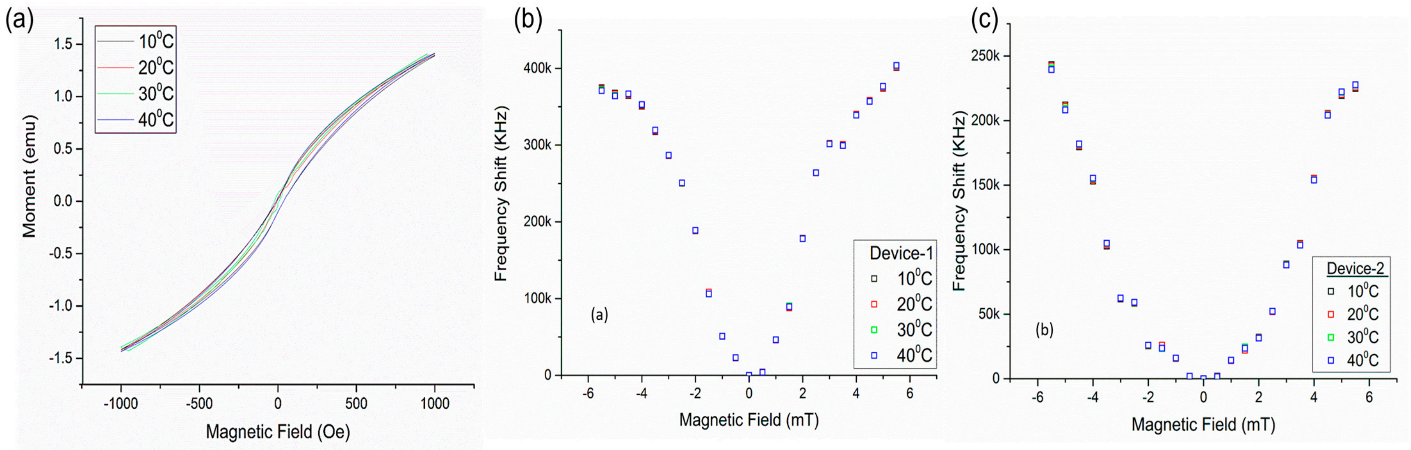

Figure S1. Temperature-dependent characteristics. (a) Magnetization curve for Terfenol-D. (b,c) Frequency shift versus magnetic field under different temperature conditions.

The actual fitting statistics:

In both Device-1 and Devive-2, Adjusted R. Squared values were found higher for sensors with Terfenol-D film grating thickness of 500 nm than the sensors with film grating thicknesses of 300 nm

and 400 nm. This implies that the response of the sensors with Terfenol-D film grating thicknesses of 500nm was closer to the ideal linear response with minimal error.

Table S1 Summary of fitting statistics and sensitivity for Device-1 and Device-2.

**Magnetic Field Range**	**Device-1**			**Device-2**		
	Adjusted R. Squared			***Adjusted R. Squared***		
	**500 nm**	**400 nm**	**300 nm**	**500 nm**	**400 nm**	**300 nm**
0 to 5mT	0.94397	0.90385	0.86531	0.94016	0.93253	0.93145
-5mT to 0	0.95488	0.91674	0.87814	0.95880	0.94499	0.94228
	Slope			***Slope***		
0 to 5mT	85.788	74.206	62.102	46.488	34.453	33.102
-5mT to 0	84.124	64.032	53.352	43.261	31.538	29.938

key performance metrics:

The resulting table below compiles key performance metrics sensitivity, limit of detection (LOD), hysteresis, and flexibility published in reputable journals between 2020 and 2026.

Table S2. Summary Table: Performance Metrics of Flexible SAW Magnetic Sensors

Sr #	***Configuration / Sensitive Layer***	***Sensitivity***	***Limit of Detection (LOD) / Field Range***	***Hysteresis***	***Flexibility / Mechanical Durability***	***Year***	***Ref***
1	*Flexible SAW magnetic sensor on $LiNbO_3$*	*forward/reverse sensitivity : 85.788 kHz/mT 0–5 mT range / 84.124 kHz/mT -5 to 0 mT range*	*0 to 5 mT (Forward) and -5 mT to 0 (Reverse)*	*Mitigated via a grating-arrayed thin film structure*	*Functional under mechanical strains of 280 με, 550 με, and 730 με (corresponding to bending radii of 89.3 mm, 45.5 mm, and 34.2 mm)*	*2026*	*This work*
2	*Love wave delay line / FeCoSiB (400 nm)*	*35 rad/T (phase)*	*Virtually constant for thickness 50–300 nm; degrades for >300 nm*	*Hysteresis losses increase with film thickness; for >300 nm, losses outweigh sensitivity gains*	*Rigid substrate (not flexible)*	*2020*	*[1]*
3	*FSMA (Ni–Mn–In) / AlN/Kapton (flexible)*	*0.94 Hz/nT*	*Not reported*	*Not reported*	*Excellent: ~200 bending cycles without resonance frequency degradation; flexible Kapton substrate*	*2024*	*[2]*
4	*Au-SiO_2 phononic crystal structure*	*Considerably improved (qualitative)*	*Not reported*	*Not reported*	*Not specified (likely rigid)*	*2025*	*[3]*
5	*FeCoSiB multilayers with exchange bias*	*Sensitivity increases with center frequency*	*55 pT/ √Hz @ 10 Hz; 9 pT/ √Hz @ 100 Hz*	*Not reported; exchange bias reduces magnetic noise*	*Rigid $LiNbO_3$ substrate*	*2025*	*[4]*
6	*Magnetostrictive FeCoSiB films*	*Not explicitly stated*	*<200 pT/ √Hz @ 10 Hz*	*Not reported*	*Rigid piezoelectric single crystal*	*2021*	*[5]*

	(RF/DC sputter)				*substrates*		
7	*Thin-film AlScN on Si / FeCoSiB*	*Not explicitly stated*	*2.4 nT/ √Hz @ 10 Hz; 72 pT/ √Hz @ 10 kHz*	*Not reported*	*Rigid Si substrate*	*2021*	*[6]*

It is worth noting that, across the surveyed literature, relatively few flexible SAW magnetic sensors report quantitative data simultaneously for all four parameters. Most flexible devices emphasize mechanical durability and proof-of-concept validation, with LOD and hysteresis often not provided. Conversely, devices exhibiting excellent LOD and low hysteresis are typically fabricated on rigid substrates. This observation does not detract from the value of existing studies but rather highlights an area where further systematic reporting would benefit the field.

Statistical Analysis and Reporting:

The following section presents the statistical analysis performed to improve the rigor and reproducibility of the experimental results.

Table S3: Experimental statistics summary

Parameter	Value
Number of devices tested	5 sets of each device
Iterations per magnetic field step	20
Total samples per data point (N)	100
Magnetic field range	±5 mT
Stabilization time per step	1 minute
Measurement type	Frequency shift (Δf)

Table S4: Sensitivity and statistical variation across devices

Device	Thickness (nm)	Sensitivity (kHz/mT)	Standard Deviation (kHz/mT)	Coefficient of Variation (%)
Device-1	300	62.4	2.8	4.49
Device-1	400	74.6	2.5	3.35
Device-1	500	85.8	2.2	2.56
Device-2	300	34.2	2.1	6.14
Device-2	400	40.8	1.9	4.65
Device-2	500	46.5	1.8	3.87

Table S5: Curve fitting quality (R^2 Analysis)

Device	Thickness (nm)	R^2 (0 to 5 mT)	R^2 (-5 to 0 mT)	Fit Quality
Device-1	300	0.991	0.988	Good
Device-1	400	0.995	0.993	Very Good
Device-1	500	0.998	0.997	Excellent
Device-2	300	0.986	0.984	Good
Device-2	400	0.991	0.989	Very Good
Device-2	500	0.995	0.993	Excellent

Table S6: Frequency stability and error analysis

Device	Max Frequency Shift (kHz)	Standard Deviation (kHz)	Relative Error (%)
Device-1 (500 nm)	377	6.5	1.72
Device-2 (500 nm)	223	5.2	2.33

Table S7: Repeatability across devices

Parameter	Device-1 (500 nm)	Device-2 (500 nm)
Mean Sensitivity (kHz/mT)	85.8	46.5
Min Sensitivity	83.9	44.8
Max Sensitivity	87.6	48.1
Spread (Max–Min)	3.7	3.3
Repeatability (%)	±2.2%	±3.5%

Quantitative Bending Analysis:

This section provides a quantitative analysis of the sensor's mechanical flexibility, including bending radius calculations and repeatability metrics. To quantitatively evaluate flexibility, the applied strain levels were converted into bending radius using classical beam theory:

$$\varepsilon = \frac{t}{2R} \Rightarrow R = \frac{t}{2\varepsilon}$$

where ε is the strain, t is the substrate thickness (50 μm), and R is the bending radius. The results are summarized below:

Table S8: Applied strain and corresponding bending radius.

Strain (με)	*Strain (ε)*	*Bending Radius (mm)*
280	*280×10^{-6}*	*89.3*
550	*550×10^{-6}*	*45.5*
730	*730×10^{-6}*	*34.2*

These values confirm stable sensor operation under bending conditions relevant to flexible and conformable electronic systems.

1. Repeatability and Statistical Reliability

Repeatability was ensured through multiple experimental iterations and device-level testing. For each magnetic field step, 20 measurements were recorded across five independently fabricated devices, yielding a total of 100 measurements per condition. This large dataset ensures statistical reliability and minimizes random noise and measurement uncertainty. The consistent response across all measurements indicates strong repeatability under mechanical deformation.

2. Sensitivity Stability and Mechanical Robustness

The sensor sensitivity is defined as:

$$S = \frac{\Delta f}{\Delta H}$$

Under mechanical stress, the total observed frequency shift can be expressed as:

$$\Delta F_{\text{total}} = \Delta F(S) + \Delta F(H)$$

Where $\Delta F(S)$ is the stress-induced frequency shift and $\Delta F(H)$ is the magnetic field-induced frequency shift. To isolate the magnetic contribution, a differential compensation method was applied:

$$\Delta F_{\text{corrected}} = \Delta F(H)$$

This approach effectively removes the influence of mechanical deformation, ensuring that the measured response corresponds solely to the applied magnetic field. Experimental results confirm that while mechanical stress introduces a frequency offset, the sensitivity remains largely unaffected after compensation, demonstrating strong mechanical robustness.

3. Scope and Future Work

We acknowledge that cyclic bending durability was not explicitly evaluated in this study. However, the consistent performance observed under multiple strain levels, combined with statistical repeatability and effective compensation, provides evidence of reliable operation under mechanical deformation. We have added a statement in the revised manuscript noting that future work will include systematic cyclic loading and long-term durability assessments.

Experimental setup for testing SAW magnetic sensors

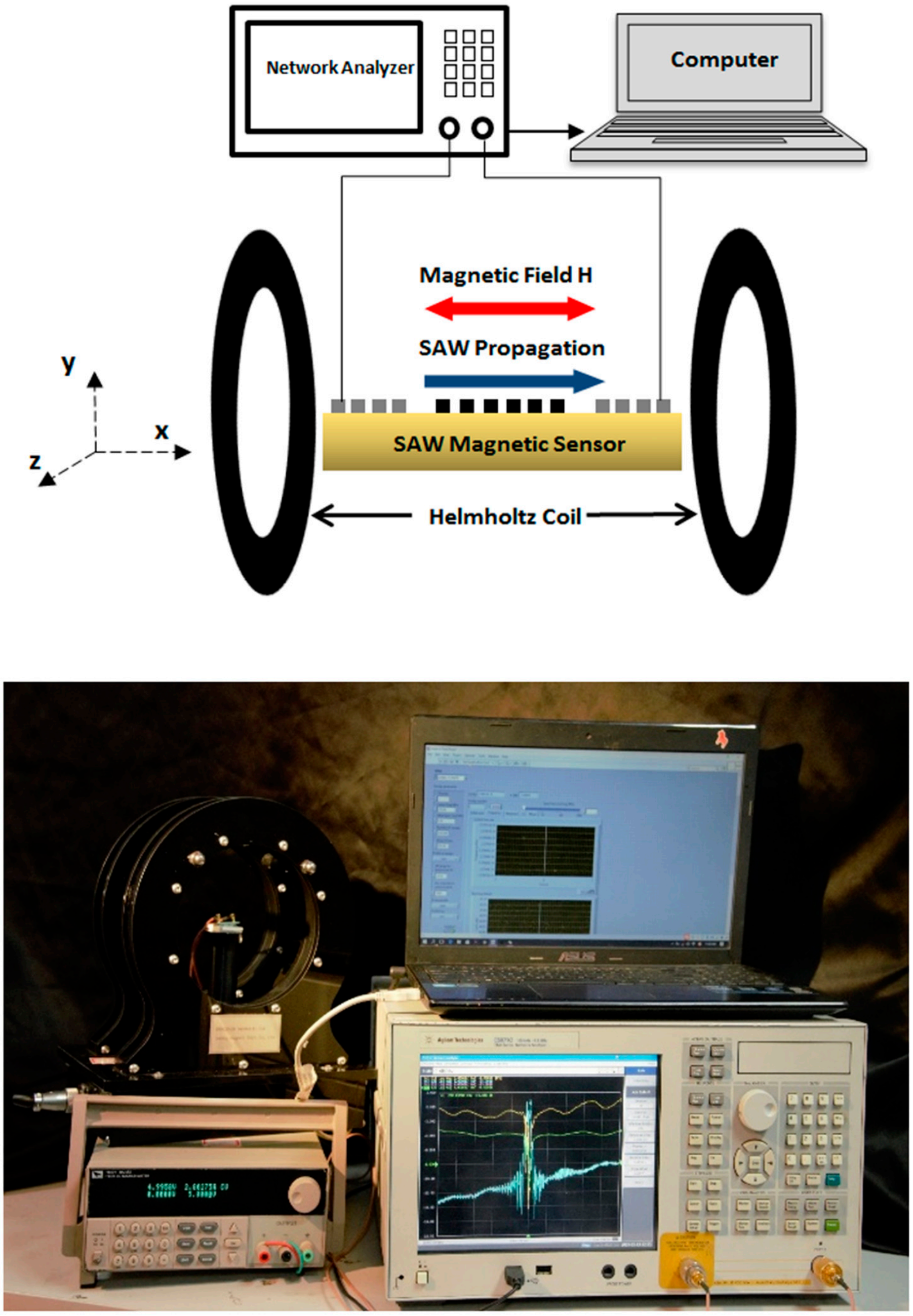

Figure S2. *Experimental setup for testing SAW magnetic sensors. The vector network analyzer (VNA) was connected to the computer via GPIB to stream data in real time to the LabVIEW program.*

Effects of stress on the measurements

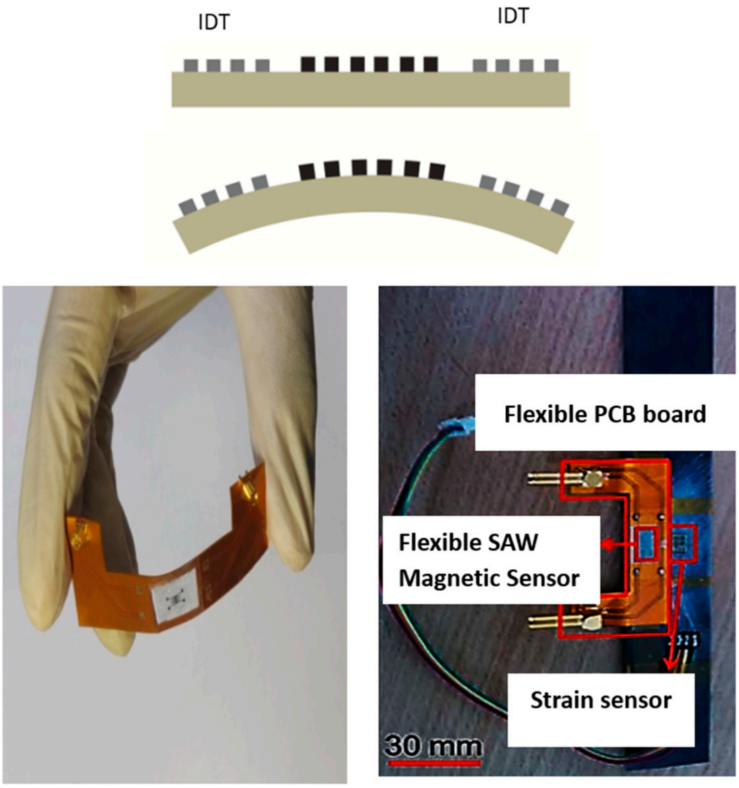

Figure S3. *Illustration of the flexible SAW magnetic sensor under mechanical stress. The sensor was mounted on a flexible PCB and subjected to controlled bending forces. Stress levels P1, P2, and P3 correspond to bending strains of* $280\,\mu\varepsilon$, $550\,\mu\varepsilon$, *and* $730\,\mu\varepsilon$, *respectively; P0 represents the unstrained reference condition.*

The dependence of SAW velocity and phase on the magnetic field arises from the magnetostrictive properties of the Terfenol-D thin film gratings used in our design. When subjected to a magnetic field, the inherent magnetostriction of Terfenol-D alters its dimensions and mechanical properties, including density and elastic coefficients, as outlined in Eq. 3 and Eq. 4. These changes induce a variation in the velocity v_{saw}, expressed as:

$v_{SAW} \propto \sqrt{\frac{c_{ijkl}}{\rho}}$ here c_{ijkl} represents the stiffness tensor (affected by ΔE due to magnetostriction), and ρ denotes the density, which is also field-dependent. The changes in velocity, in turn, affect the phase ϕ of the SAW, as given by:

$$\phi = \frac{2\pi f L}{v_{SAW}}$$

where f is the frequency and L is the propagation length. The application of the magnetic field modifies both c_{ijkl} and ρ, leading to measurable shifts in both the velocity and phase. These shifts are captured in our experimental setup by monitoring the frequency shifts, which are a direct consequence of the phase change and can be correlated back to the applied magnetic field.

References:

1. Kittmann, A.; Müller, C.; Durdaut, P.; Thormählen, L.; Schell, V.; Niekiel, F.; Lofink, F.; Meyners, D.; Knöchel, R.; Höft, M.; et al. Sensitivity and noise analysis of SAW magnetic field sensors with varied magnetostrictive layer thicknesses. *Sensors and Actuators A: Physical* **2020**, *311*, 111998, doi:https://doi.org/10.1016/j.sna.2020.111998.
2. Kumar, P.; Singh, J.; Kaur, D. Ferromagnetic Shape Memory Alloy Integrated Highly Flexible SAW Delay Line Magnetic Sensor. *IEEE Sensors Journal* **2024**, *24*, 2664-2670, doi:10.1109/JSEN.2023.3345031.
3. Samadi, M.; Meyer, J.M.; Spetzler, E.; Spetzler, B.; McCord, J.; Lofink, F.; Gerken, M. Modeling of High-Sensitivity SAW Magnetic Field Sensors with Au-SiO2 Phononic Crystals. *Advanced Sensor Research* **2025**, *4*, 2500008, doi:https://doi.org/10.1002/adsr.202500008.
4. Weisheit, F.; Wolframm, H.; Malavé, A.; Höft, M.; Quandt, E.; Meyners, D. Frequency tuning and sensitivity enhancement of surface acoustic wave sensors for magnetic field detection. *Sensors and Actuators A: Physical* **2026**, *398*, 117316, doi:https://doi.org/10.1016/j.sna.2025.117316.
5. Thormählen, L.; Seidler, D.; Schell, V.; Munnik, F.; McCord, J.; Meyners, D. Sputter Deposited Magnetostrictive Layers for SAW Magnetic Field Sensors. *Sensors* **2021**, *21*, 8386.
6. Meyer, J.M.; Schell, V.; Su, J.; Fichtner, S.; Yarar, E.; Niekiel, F.; Giese, T.; Kittmann, A.; Thormählen, L.; Lebedev, V.; et al. Thin-Film-Based SAW Magnetic Field Sensors. *Sensors* **2021**, *21*, 8166.
